# Overcoming barriers to reprogramming and differentiation in nonhuman primate induced pluripotent stem cells

**DOI:** 10.5194/pb-4-153-2017

**Published:** 2017-08-18

**Authors:** Jacob J. Hemmi, Anuja Mishra, Peter J. Hornsby

**Affiliations:** Barshop Institute and Department of Physiology, University of Texas Health Science Center San Antonio, San Antonio, TX 78245, USA

## Abstract

Induced pluripotent stem cells (iPS cells) generated by cellular reprogramming from nonhuman primates (NHPs) are of great significance for regenerative medicine and for comparative biology. Autologously derived stem cells would theoretically avoid any risk of rejection due to host–donor mismatch and may bypass the need for immune suppression post-transplant. In order for these possibilities to be realized, reprogramming methodologies that were initially developed mainly for human cells must be translated to NHPs. NHP studies have typically used pluripotent cells generated from young animals and thus risk overlooking complications that may arise from generating iPS cells from donors of other ages. When reprogramming is extended to a wide range of NHP species, available donors may be middle- or old-aged. Here we have pursued these questions by generating iPS cells from donors across the life span of the common marmoset (*Callithrix jacchus*) and then subjecting them to a directed neural differentiation protocol. The differentiation potential of different clonal cell lines was assessed using the quantitative polymerase chain reaction. The results show that cells derived from older donors often showed less neural marker induction. These deficits were rescued by a 24 h pretreatment of the cells with 0.5 % dimethyl sulfoxide. Another NHP that plays a key role in biological research is the chimpanzee (*Pan troglodytes*). iPS cells generated from the chimpanzee can be of great interest in comparative in vitro studies. We investigated if similar deficits in differentiation potential might arise in chimpanzee iPS cells reprogrammed using various technologies. The results show that, while some deficits were observed in iPS cell clones generated using three different technologies, there was no clear association with the vector used. These deficits in differentiation were also prevented by a 24 h pretreatment with 0.5 % dimethyl sulfoxide.

## 1 Introduction

Induced pluripotent stem cells (iPS cells) are the equivalent of embryonic stem cells yet are derived from somatic cells by reprogramming (Takahashi and Yamanaka, 2006; Takahashi et al., 2007). The first nonhuman primate (NHP) iPS cells were from the rhesus macaque (Liu et al., 2008); the second NHP species to be reprogrammed was the common marmoset, reported by this lab in 2010 (Wu et al., 2010). Because of the genetic and physiological relatedness of NHPs to humans, NHP iPS cells have a particular importance. In particular, as a development in regenerative medicine, they may be critical to solving the issue of whether autologous cells (cells derived from the donor and reprogrammed to a cell type suitable for therapeutic use) are superior in their properties to allogeneic cells (Qiu et al., 2013). While these questions could be addressed in rodents or other species, the special place in biology of NHPs makes them ideal for a definitive answer to this question. Moreover, the availability of NHP iPS cells creates opportunities for explorations of comparative biology in NHP species that are inaccessible or unsuitable for biomedical research, such as the chimpanzee (Wunderlich et al., 2014).

One of the critical questions that relates to the potential use of autologous cell therapy is whether reprogramming of somatic cells from donors other than newborns or young animals yields cells that are of the same quality and utility as iPS cells derived from young donors. This is of particular importance considering that cells available from different NHP species may only be from middle- or old-aged donors. Comparative studies of differentiated cell types derived from iPS cells may be very valuable for understanding many aspects of primate biology. Moreover, it is the older part of the human population that is the main target for potential iPS-cell-based therapy. Cell therapy is typically considered to be of major importance in chronic degenerative diseases of aging (Qiu et al., 2013). In a sense, the issue is whether such older cells can be “rejuvenated” by reprogramming (Lapasset et al., 2011) or whether they may retain age-related defects that render them less useful for therapeutic purposes.

Cellular reprogramming is the process by which terminally differentiated cells are converted into stem cells. This was first achieved by somatic cell nuclear transfer (Gurdon, 1962) and about a decade ago through forced expression of key transcription factors (Takahashi and Yamanaka, 2006). Stem cells derived through reprogramming are known as induced pluripotent stem cells and share many, if not all, properties with embryonic stem (ES) cells, including the ability to generate all the tissues and organs in the adult body (Choi et al., 2016). Autologous cell therapy, based on iPS cell technology, also holds out the possibility of reducing or eliminating the need for immunosuppression after transplantation, as the transplanted cells will be a complete genetic match for the recipient (Qiu et al., 2013).

**Figure 1:**
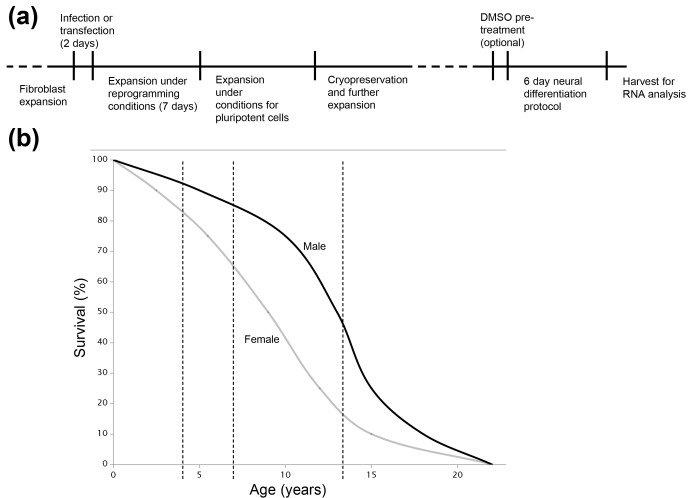
**(a)** Timeline showing the transition of terminally differentiated cells (fibroblasts) first to iPS cells, through reprogramming, and then to neural type cells using a directed differentiation method. **(b)** Life span curve of the common marmoset in a closed colony setting. Curve derived by smoothing the graphs reported by Nishijima et al. (2012). Dashed vertical lines indicate the ages of marmoset donors used to generate iPS cells in these studies. The survival pattern in the SNPRC colony from which our samples were derived is very similar to that shown here (Ross et al., 2012).

Although experiments have primarily been done using iPS cells derived from young animals or human subjects, various investigations have addressed the effect of cellular changes associated with aging with regard to reprogramming efficiency (Mahmoudi and Brunet, 2012), mitochondrial structure (Prigione et al., 2010), telomere length (Yu et al., 2007), epigenetic memory (Polo et al., 2010) and somatic mutations (Sardo et al., 2017). To our knowledge, no study to date has addressed the differentiation potential of iPS cells generated from aged versus young NHP donors.

In these experiments we focused on one of the many NHP species in use in biomedical research, the common marmoset (*Callithrix jacchus*). This species was selected for numerous reasons. They have a relatively short life span for a primate, and they are more easily maintained under laboratory conditions than other NHPs used in biomedical research (Mattison and Vaughan, 2016). Our lab has expertise in the generation of iPS cells from marmosets (Wu et al., 2010) and experience in directed neural differentiation protocols optimized for marmoset iPS cells (Farnsworth et al., 2013; Qiu et al., 2015) (Fig. 1a). The use of NHPs versus more common rodent models is of special importance in view of the fact that mouse iPS cells represent a different state of pluripotency with distinct properties not shared by iPS cells generated from species such as human or marmoset (Nichols and Smith, 2009). We had access to somatic cells from marmosets of various ages – in particular to animals that are relatively old (13 years), representing about the 30 % survival point of the marmoset life span in the Southwest National Primate Research Center (SNPRC, San Antonio, TX, USA) colony (Fig. 1b). Our second aim was to generate iPS cells from the chimpanzee, as a model in vitro system for comparative studies with humans and other NHPs. While chimpanzee iPS cells have been described (Marchetto et al., 2013; Fujie et al., 2014), they have been much less studied than those from other NHP species. We were interested in determining what the optimal method for generating chimpanzee iPS cells may be. In both cases, cells derived from aged marmosets and cells derived by different methods from chimpanzee cells, we encountered barriers to appropriate differentiation that could be overcome in most cases by prior treatment with dimethyl sulfoxide (DMSO). DMSO pretreatment is a technique that was originally described to optimize the differentiation of human pluripotent cells to insulin-secreting cells (Chetty et al., 2013) and was found by our group to be valuable in eliminating clonal variation in differentiation potential for NHP cells (Qiu et al., 2015).

## 2 Methods

### 2.1 Induction of pluripotency

Most methods used in these experiments have been described in detail in a recent book chapter (Mishra et al., 2016) and are summarized here in Fig. 1a. The starting somatic cells were skin fibroblasts. Skin samples were obtained from a newborn female marmoset, a 4-year-old male, a 7-year-old female and two different 13-year-old male animals. All samples were obtained from tissue samples of animals being euthanized at the SNPRC . The chimpanzee skin sample was obtained in 2011 from a stillborn animal, also at the SNPRC. Fibroblasts were derived from the skin samples as previously described (Mishra et al., 2016).

For all marmoset cell experiments and for derivation of chimpanzee iPS cells using retroviral vectors, fibroblasts were trypsinized and plated in polylysine-coated six-well plates (Corning, Tewksbury, MA, USA). The day following plating, cells were infected with a mixture of four pMXs retroviruses, each encoding one of the reprogramming factors OCT4, SOX2, KLF4 and c-MYC (Salk Institute GT3 core), and the infection was repeated once more the following day as previously described (Wu et al., 2009). For chimpanzee Sendai iPS clones, infection was carried out using the CytoTune-iPS Sendai Reprogramming Kit according to the manufacturer's protocol (Invitrogen). Chimpanzee Epstein–Barr virus nuclear antigen (EBNA) episomal iPS clones were generated using the Episomal iPSC Reprogramming Vectors (Invitrogen) according to the manufacturer's protocol. The plasmids were introduced into the fibroblasts via electroporation using a Nucleofector instrument (Lonza). Following infection or transfection, cells were treated over the next several weeks as previously described and as shown in outline in Fig. 1. Routine iPS cell maintenance was in E8 medium (Chen et al., 2011) supplemented with 10 % fetal bovine serum (GlobalStem, Gaithersburg, MD, USA). Culture plates were coated with Matrigel. Subculturing was performed with Accutase (BioExpress, Kaysville, UT, USA).

### 2.2 Directed neural differentiation

All iPS cell clones were grown in E8 medium until the cells were to be tested for their differentiation potential. As the cells approached confluency, in the 24 h preceding the protocol cells were incubated in E8 medium either with or without 0.5 % DMSO (Sigma Aldrich, St. Louis, MO, USA). The following day the medium was aspirated, and the cells were disassociated with Accutase. Cells were collected, centrifuged and suspended at a concentration of 3000 cells per 30 µL in the differentiation medium previously described (Qiu et al., 2015). The lid of a 96-well plate was used to create numerous 30 µL droplets, each containing 3000 cells. The lids were then inverted and placed in a humidified incubator at 37.5${}^{\circ }$ C for 72 h and allowed to form embryoid bodies. After this time the cells were collected by flushing the lids with DMEM/F12 into a 15 mL conical tube and allowed to settle under gravity. The medium was then aspirated, and the embryoid bodies were resuspended in 2 mL of a second differentiation medium, also as previously described (Qiu et al., 2015), and plated in a nonadherent 35 mm dish. Cells were maintained in the same medium for 72 h. Following this period, cells were harvested for preparation of RNA.

**Table 1:** Expression of pluripotency genes *NANOG* and *OCT4* in marmoset iPS cell clones. The designation of the clones – derived from a newborn, a 4-year-old, a 7-year-old and two 13-year-old marmosets – is as described in the text. The values are given as Ct(gene) – Ct(β-actin). Note that lower numerical values represent higher mRNA levels.

		Newborn	4 yo	7 yo	13 yo 1A	13 yo 1B	13 yo 1C	13 yo 2A	13 yo 2B	13 yo 2C
*NANOG*	Mean	7.5	5.8	7.1	7.6	8.1	6.4	6.4	5.6	6.1
	SD	0.5	1.4	0.9	0.4	1.1	0.6	1.2	0.9	0.6
*OCT4*	Mean	2.0	1.5	2.8	2.4	2.1	2.5	3.7	1.3	1.0
	SD	0.6	0.9	1.0	1.0	0.7	1.2	0.5	0.4	1.6

### 2.3 qPCR

Total RNA was isolated from cells using RNA Bee (Tel-Test, Friendswood, TX, USA) according to the manufacturer's instructions. A total of 2 µg of RNA was reverse-transcribed by using SuperScript II (Life Technologies). Quantitative polymerase chain reaction (qPCR) was conducted using SYBR Green detection and an ABI 7900HT system (Applied Biosystems, Foster City, CA, USA). Levels of messenger RNAs (mRNAs) are reported as cycles versus β-actin. The data analysis for this paper was generated using Microsoft Excel and the Real Statistics Resource Pack software (Release 4.3; copyright 2013–2015 Charles Zaiontz, www.real-statistics.com). Responses to differentiation and DMSO pretreatment were analyzed by one-way analysis of variance (ANOVA) followed by Dunnett's test of multiple comparisons with a control.

## 3 Results

### 3.1 Reprogramming of cells from marmosets of different ages

**Figure 2:**
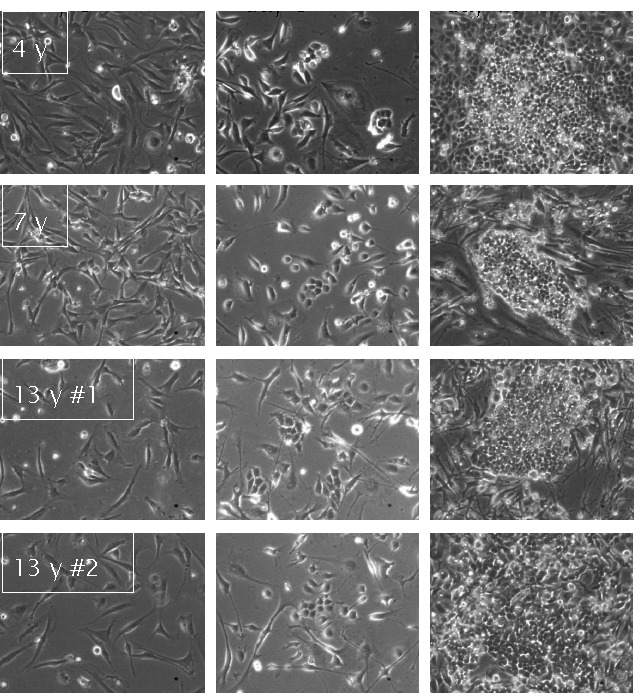
Development of iPS colonies generated from a 4-year-old, a 7-year-old and two 13-year-old marmosets. Columns from left to right show colony development at 0 days, 8 days and 11 days after the second retroviral infection. Generated iPS cells quickly displace surrounding fibroblasts as tight colonies develop. The characteristic morphology of the newly formed colonies is a good indicator of reprogramming.

For this study we selected animals of a range of ages spanning most of the life span of the marmoset (a newborn female, a 4-year-old male, a 7-year-old female and two 13-year-old male animals). We chose the 13-year-old animals as representing individuals within the senescent period of the life span of the marmosets in this colony (Fig. 1b). Multiple clones were derived from the older animals in order to test the possibility that there are defects in differentiation in some iPS cells derived from older donors. iPS cells were generated by forced expression of the Yamanaka reprogramming factors OCT4, SOX2, KLF4 and c-MYC by retroviral transduction (Fig. 1a). Colonies began to appear around day 8 and were clearly discernible and large enough for isolation by day 11 at all ages (Fig. 2a). To confirm proper reprogramming, we performed qPCR on the isolated clones to determine the expression levels of the pluripotency markers NANOG and endogenous OCT4. We found appropriate expression levels for all clones derived. In all cases the levels were much higher than those in skin fibroblasts, as previously reported (Wu et al., 2010). However there were more minor differences in expression of these pluripotency factors among the different iPS cell clones (Table 1) The frequency of iPS cell clone generation, colony formation and the rate of cell growth were all unaffected by donor age. Cellular morphology of all of the derived iPS cell clones was as expected for marmoset iPS cells (Fig. 2). The distinctive morphology (cell shape, nuclear / cytoplasmic ratio, cell attachment in colonies) has been shown to be a good indicator of reprogramming in marmoset iPS cells (Wu et al., 2010) and in pluripotent cells generally (Quintanilla Jr., 2013).

**Figure 3:**
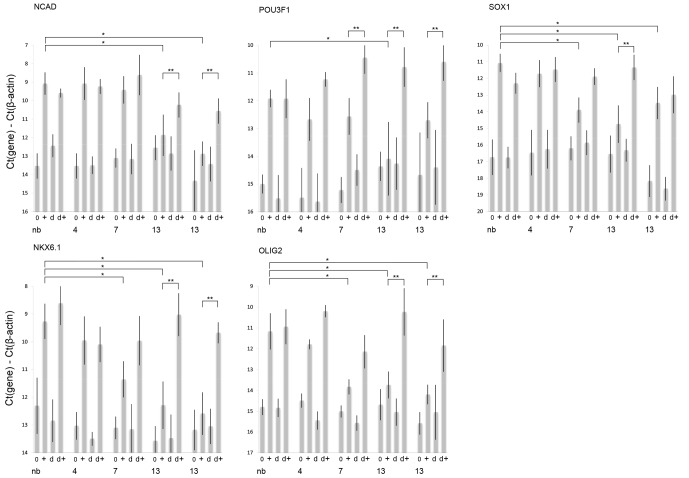
mRNA expression levels for the neural differentiation markers NCAD, POU3F1, SOX1, NKX6.1 and OLIG2 as measured by qPCR. Bars are grouped by marmoset donor ages: newborn (nb), 4 (4 years old), 7 (7 years old) and 13 (each of the two 13-year-olds). Labels along the x axis indicate treatment groups as follows: cells that have not been pretreated with 0.5 % DMSO are marked 0 before differentiation and + after the differentiation protocol. Cells that have been pretreated with 0.5 % DMSO are marked d before differentiation and d+ after the differentiation protocol. A single asterisk indicates that the cells in this group display significantly lower marker expression (p < 0.05 one-way ANOVA followed by Dunnett's test) than newborn controls that were subjected to the differentiation protocol but not pretreated with DMSO. A double asterisk indicates that the cells in this group, when differentiated after pretreatment with DMSO, display significantly increased marker expression (p<0.05 one-way ANOVA followed by Dunnett's test) when compared with their same-age controls that were not pretreated with DMSO. Each directed differentiation was replicated three times per clone and averaged (bars for nb, 4 and 7: n=3; three clones were generated for each 13-year-old animal; bars for both animals: n=9).

**Figure 4:**
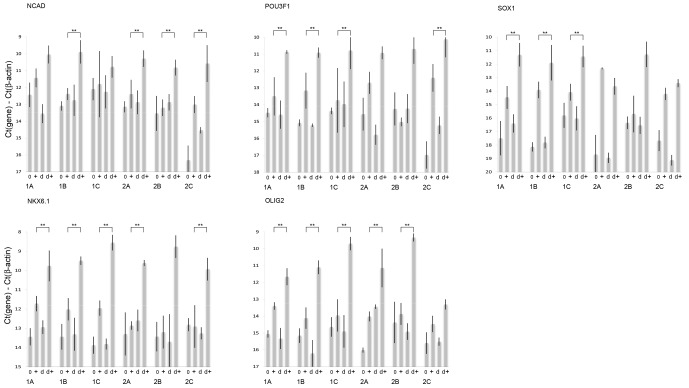
mRNA expression levels for the neural differentiation markers NCAD, POU3F1, SOX1, NKX6.1 and OLIG2 as measured by qPCR. Each grouping of bars represents an iPS clone generated from a 13-year-old marmoset. Groups 1A, 1B and 1C represent three separate clones generated from a single 13-year-old, while groups 2A, 2B and 2C represent three separate clones generated from the other 13-year-old. Labels along the x axis indicate what treatment groups the bar represents. Cells that have not been pretreated with 0.5 % DMSO are marked 0 before differentiation and + after the differentiation protocol. Cells that have been pretreated with 0.5 % DMSO are marked d before differentiation and d + after the differentiation protocol. A double asterisk indicates that the cells in this group, when differentiated after pretreatment with DMSO, display significantly increased marker expression (p<0.05 one-way ANOVA followed by Dunnett's test) when compared with their same clone controls that were not pretreated with DMSO. Each directed differentiation was replicated three times per clone and averaged: n=3.

### 3.2 Directed differentiation of iPS cells from marmosets of different ages

We selected one iPS cell clone each from the newborn, 4-year-old and 7-year-old animals and three clones from each of the 13-year-old animals. Cells of each clone were subjected to a directed neural differentiation protocol previously employed in this lab (Qiu et al., 2015). After 7 days the differentiated cells were assayed for the expression levels of NCAD, POU3F1, SOX1, NKX6.6 and OLIG2 by qPCR. These genes were previously shown to be highly responsive to the differentiation protocol used and to provide useful markers of neural differentiation generally (Fig. 3a–e). They represent a collection of marker genes that are broadly useful in assessing differentiation potential, rather than differentiation into a specialized neural lineage. The results show that NCAD expression was significantly decreased (p < 0.05) in the average of the three clones from each of the 13-year-old marmosets when compared to the newborn. For POU3F1 expression was significantly decreased in the average of the three clones from 13-year-old marmoset #1 but not 13-year-old marmoset #2 when compared to the newborn. For SOX1 expression was significantly decreased in the clone from the 7-year-old and in the average of the three clones from each of the 13-year-old marmosets when compared to the newborn. For NKX6.1 expression was significantly decreased in the clone from the 7-year-old and in the average of the three clones from each of the 13-year-old marmosets when compared to the newborn. Furthermore, for OLIG2, a significant decrease was found in the clone from the 7-year-old and in the average of the three clones from each of the 13-year-old marmosets when compared to the newborn. Because we had previously shown that 24 h DMSO pretreatment may increase the differentiation potential of marmoset iPS cells, as well as human pluripotent cells (Chetty et al., 2013; Qiu et al., 2015), we repeated our differentiation protocol after allowing the cells to incubate for 24 h with 0.5% DMSO added to the normal growth medium. The results show that after pretreatment the clone from the 7-year-old showed a significant increase in POU3F1 expression compared to the same cells without pretreatment. The iPS cells from 13-year-old marmoset #1 showed significant increases for all five markers, while 13-year-old #2 showed a significant increase for four of them.

We next investigated the six clones generated from the two 13-year-old marmosets individually (Fig. 4a–e). The results show that NCAD expression was significantly increased by DMSO pretreatment in four out of the six clones; POU3F1 expression was significantly increased by DMSO pretreatment in four out of the six clones; SOX1 expression was significantly increased by DMSO pretreatment in four of the six clones; NKX6.1 expression was significantly increased by DMSO pretreatment in five out of the six clones; and for OLIG2 a significant increase by DMSO pretreatment was found for five of the six clones from the two 13-year-old marmosets.

Overall, these experiments show that generation of iPS cell clones was feasible in the case of all ages of marmosets tested and that differentiation defects, which were apparent as a function of age in the case of some of the derived cell clones, were mostly corrected by prior treatment with DMSO. While there were minor differences in the pluripotency characteristics of the clone derived from old animals, these did not correlate with the occurrence of defects in directed differentiation.

**Figure 5:**
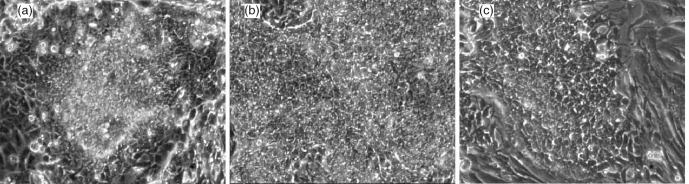
Representative images of chimpanzee iPS clones generated by **(a)** retrovirus, **(b)** Sendai virus and **(c)** episomal EBNA vector. Images were taken ∼ 25–30 days after the final infection or transfection. After colonies have been isolated onto mitomycin-treated mouse embryo fibroblasts, they expand rapidly and acquire typical pluripotent cell morphology.

### 3.3 Generation of chimpanzee iPS cells using different vectors

The chimpanzee is a NHP species of exceptional interest, and iPS cells from this species will be of great value in comparative in vitro studies. Many technologies are currently available for the generation of iPS cells. In order to investigate what effect vector choice might have on the differentiation potential of chimpanzee iPS cells, we generated multiple iPS cell clones using three different technologies: an integrating retrovirus, an RNA-based replicative virus (Sendai virus) and a replicating episomal plasmid vector based on the Epstein–Barr virus genome (EBNA). All three vectors were able to generate multiple clones in approximately the same time frame and with protocols similar to that of the previously mentioned retroviral generation method (Fig. 1a). In place of the retroviral infection step, cells were exposed to recombinant Sendai virus vectors or were transfected by electroporation with the episomal EBNA vectors. Upon isolation, clones from all vectors displayed normal growth characteristics and displayed typical morphologies (Fig. 5).

### 3.4 Differentiation of chimpanzee iPS cells using different vectors

**Figure 6:**
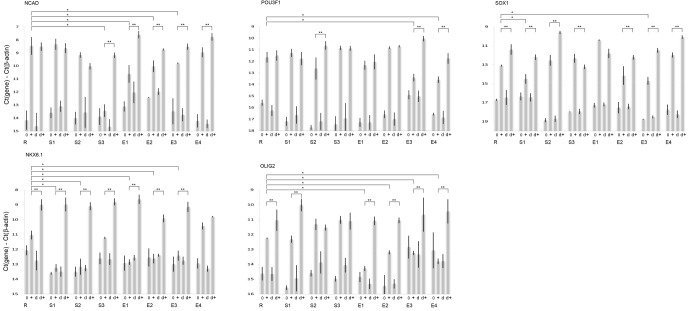
mRNA expression levels for the neural differentiation markers NCAD, POU3F1, SOX1, NKX6.1 and OLIG2 as measured by qPCR. Bars are grouped by which vector was used to produce each chimpanzee iPS clone: retrovirus (R), Sendai virus (S1, S2 and S3) or episomal EBNA vector (E1, E2, E3 and E4). Labels along the x axis indicate the treatment groups. Cells that have not been pretreated with 0.5 % DMSO are marked 0 before differentiation and + after the differentiation protocol. Cells that have been pretreated with 0.5 % DMSO are marked d before differentiation and d + after the differentiation protocol. A single asterisk indicates the cells in this group display significantly lower marker expression (p<0.05 one-way ANOVA followed by Dunnett's test) than the retrovirally generated clone subjected to the differentiation protocol but not pretreated with DMSO. A double asterisk indicates that the cells in this group, when differentiated after pretreatment with DMSO, display significantly increased marker expression (p<0.05 one-way ANOVA followed by Dunnett's test) when compared with their same clone controls that were not pretreated with DMSO. Each directed differentiation was replicated three times per clone and averaged: n=3.

We then exposed the chimpanzee iPS cells generated from each vector to the same neural differentiation protocol as used for marmoset iPS cells. We assayed the same neural markers to quantify the level of differentiation exhibited by each clone (Fig. 6a–e). In each case we compared the extent of induction of the same differentiation markers as used for the marmoset cells and compared the induction with that observed in the retrovirally reprogrammed cells as our standard. For NCAD we found significantly decreased expression in one out of three Sendai virus clones and three out of four EBNA clones when compared to a retrovirally generated clone. For POU3F1 we found significantly decreased expression in none of three Sendai virus clones and two out of four EBNA clones when compared to a retrovirally generated clone. For SOX1 we found significantly decreased expression in one of three Sendai virus clones and one out of four EBNA clones when compared to a retrovirally generated clone. For NKX6.1 we found significantly decreased expression in two out of three Sendai virus clones and three out of four EBNA clones when compared to a retrovirally generated clone. Finally, for OLIG2 we found significantly decreased expression in none of three Sendai virus clones and four out of four EBNA clones when compared to a retrovirally generated clone. We once again repeated the differentiation with a 24 h pretreatment of 0.5 % DMSO in the growth medium and found that many of the markers that had previously shown previous decreases were now significantly increased. For NCAD we found significantly increased expression in one out of three Sendai virus clones and four of four EBNA clones when compared to differentiation without pretreatment. For POU3F1 we found significantly increased expression in one of three Sendai virus clones and two out of four EBNA clones when compared to differentiation without pretreatment. For SOX1 we found significantly increased expression in three out of three Sendai virus clones and three of four EBNA clones when compared to differentiation without pretreatment. For NKX6.1 we found significantly increased expression in the retroviral clone, one out of three Sendai virus clones and three out of four EBNA clones when compared to differentiation without pretreatment. Finally, for OLIG2 we found significantly increased expression in the retroviral clone, one out of three Sendai virus clones and four out of four EBNA clones when compared to differentiation without pretreatment.

Overall, therefore, we showed that chimpanzee iPS cells may be generated by multiple technologies and that clone-specific defects in differentiation under standardized conditions can be mostly eliminated by prior exposure to DMSO.

## 4 Discussion

Given the important role of NHPs in regenerative medicine, characterizing NHP iPS cells and defining barriers to reprogramming and differentiation are crucial. Here we report the successful generation of iPS clones from marmosets of various ages, spanning most of the life span of this species. There was no difficulty in obtaining pluripotent cell clones from older donors. However, we show that upon directed differentiation in the neural pathway some clones from older donors showed significantly lower expression of neural markers. Interestingly this was not correlated with the level of expression of pluripotency markers in these iPS cell clones. Pretreatment comprising incubation with 0.5 % DMSO for 24 h before the differentiation protocol corrected the deficit in many cases. In particular this procedure robustly improved the differentiation potential of iPS cell clones obtained from the oldest (13-year-old) animals. While the generalizability of this finding is yet to be determined, the implication is that such a protocol could be routinely employed in autologous cell therapy experiments in NHP species, particularly when the donors are in the older age range. Typically in an autologous experiment, there is a limited window of time between taking the initial cell sample and the time scheduled for the cell therapy experiment. Any procedure that enables the derivation of pluripotent cells and their differentiation to a therapeutic cell type to be more robust is very valuable. Instead of extensive screening of large numbers of clones for the small percentage that may show good differentiation in the absence of this pretreatment, routine use of DMSO could greatly enhance the value of such translational models.

Because of the extreme difficulty in studying differentiated cell types from nonhuman primates, the availability of iPS cells from a range of species is of extraordinary utility for studies of comparative biology. Many of these species are not available as research subjects, either due to their scarcity or due to regulatory limitations. It was therefore of great interest that we found that DMSO pretreatment erased most of the intraclonal variation in differentiation of iPS cell clones derived from newborn chimpanzee fibroblasts. There is great interest in the in vitro use of chimpanzee iPS cells in comparative studies, particularly in neuroscience. The ability to select the most convenient reprogramming method, including completely nonviral methods based on episomal plasmids, is of great value. Possible intraclonal variations in differentiation potential may be addressed by prior DMSO pretreatment. Given these results, future studies on derivation and differentiation of chimpanzee iPS cells may include more use of nonviral, nonintegrating vectors such as those shown to be effective here. Chimpanzee iPS cells represent the only currently available method for deriving and studying specialized cell types in this species. In particular, the use of chimpanzee iPS cells in comparative studies of neural function has already given significant insights into differences in neural function between chimpanzees and humans (Marchetto et al., 2013). This is an area in which considerable expansion of the scope of research in the future may be anticipated, due to (1) continued improvement of methods for deriving accurate in vitro models of organ function from pluripotent stem cells and (2) expansion of the range of iPS cells from nonhuman primate species, enabling increasingly sophisticated comparative studies (Wunderlich et al., 2014).

We investigated the possible effect of age of donor on the differentiation properties of reprogrammed clones of cells. The ability to create iPS cells from older human donor cell samples has been well established over the past 10 years, and therefore our ability to do the same in the marmoset was anticipated. However, the varieties of subtle defects in differentiation or other changes in iPS cells derived from older donors are still an area of very active investigation. Networks of transcription factors that maintain a specific differentiated state can be disrupted during reprogramming and reset to an embryonic state; this was the initial hypothesis tested during the first reprogramming experiments, and the validity of this process as the basis of reprogramming has been extensively validated (Takahashi and Yamanaka, 2006). However, the ability to reprogram cells by the forced expression of transcription factors may not be limitless (Polo et al., 2010). Nuclear mutations accumulate as a function of donor age and of course cannot be reversed by reprogramming (Sardo et al., 2017). Telomere length is restored by reprogramming (Yu et al., 2007). Age-related mitochondrial genome mutations (Kazachkova et al., 2013) cannot be reversed by reprogramming, but mitochondrial heteroplasmy, which is age-related, could be subject to alterations (Prigione et al., 2010). While methylation changes in DNA are well established in aging, the methylation state of certain genes may be resistant to reprogramming; moreover, certain changes in heterochromatin that are diagnostic of the senescent state may also be resistant (Mahmoudi and Brunet, 2012). For all these reasons, while reprogramming of cells from older donors is clearly quite efficient (Lapasset et al., 2011), the resultant iPS cells may carry age-related defects that were not reversed by the reprogramming process. Overall therefore, the ability to derive iPS cells from older donors must be balanced against potential defects in differentiation when iPS cells are used as a model system for the derivation of specific differentiated cell types.

In the present experiments we observed both age-related defects (in marmoset iPS cells) and clonal variation as a function of reprogramming technology (in chimpanzee iPS cells). In a wide variety of cases these defects in differentiation could be overcome by prior pretreatment of the cells with 0.5 % DMSO. These data amplify our previous observations that DMSO can rescue clonal defects in differentiation in marmoset iPS cells (Qiu et al., 2015) and build on earlier observations of the effect of DMSO in human pluripotent cells (Chetty et al., 2013). The mode of action of DMSO in enhancing the responsiveness of pluripotent cells to inducers of differentiation is unknown. Its mechanism has been studied in detail in P19 embryonal carcinoma cells, which have the ability to differentiate into cells of the three germ layers (Choi et al., 2015). In those studies it was shown to modulate the activity of Wnt/TGF-β signalling. Despite multiple hypotheses about how it may have such actions at a molecular level (cell cycle regulation, apoptosis regulation, chromatin modification, scavenging of oxygen radicals), it cannot be replaced by other agents that have similar effects. Despite a lack of clarity on how it acts at a molecular level, it is nevertheless a very valuable tool in pluripotent cell studies.

## Supplement

10.5194/pb-4-153-2017-supplement The supplement related to this article is available online at: https://doi.org/10.5194/pb-4-153-2017-supplement.

## Data Availability

The data in Figs. 3, 4 and 6 are qPCR data (Ct values originating from the PCR machine) and can be found in the Supplement.

## References

[bib1.bib1] Chen G, Gulbranson DR, Hou Z, Bolin JM, Ruotti V, Probasco MD, Smuga-Otto K, Howden SE, Diol NR, Propson NE, Wagner R (2011). Chemically defined conditions for human iPSC derivation and culture. Nat Methods.

[bib1.bib2] Chetty S, Pagliuca FW, Honore C, Kweudjeu A, Rezania A, Melton DA (2013). A simple tool to improve pluripotent stem cell differentiation. Nat Methods.

[bib1.bib3] Choi J, Lee S, Clement K, Mallard W, Tagliazucchi GM, Lim H, Choi IY, Ferrari F, Tsankov A, Pop R, Lee G, Rinn J, Meissner A, Park PJ, Hochedlinger K (2016). A comparison of genetically matched cell lines reveals the equivalence of human iPSCs and ESCs. Nat Biotechnol.

[bib1.bib4] Choi SC, Choi JH, Cui LH, Seo HR, Kim JH, Park CY, Joo HJ, Park JH, Hong SJ, Yu CW, Lim DS (2015). Mixl1 and Flk1 and key players of Wnt/TGF-
β
signalling during DMSO-induced differentiation in P19 cells. J Cell Physiol.

[bib1.bib5] Farnsworth SL, Qiu Z, Mishra A, Hornsby PJ (2013). Directed neural differentiation of induced pluripotent stem cells from non-human primates. Exp Biol M.

[bib1.bib6] Fujie Y, Fusaki N, Katayama T, Hamasaki M, Soejima Y, Soga M, Ban H, Hasegawa M, Yamashita S, Kimura S, Suzuki S, Matsuzawa T, Akari H, Era T (2014). New type of Sendai virus vector provides transgene-free iPS cells derived from chimpanzee blood. PLoS One.

[bib1.bib7] Gurdon JB (1962). The Developmental capacity of nuclei taken from intestinal epithelium cells of feeding tadpoles. J Embryol Exp Morph.

[bib1.bib8] Kazachkova N, Ramos A, Santos C, Lima M (2013). Mitochondrial DNA damage patterns and aging: Revising the evidences for humans and mice. Aging and Disease.

[bib1.bib9] Lapasset L, Milhavet O, Prieur A, Besnard E, Babled A, Aït-Hamou N, Leschik J, Pellestor F, Ramirez JM, De Vos J, Lehmann S (2011). Rejuvenating senescent and centenarian human cells by reprogramming through the pluripotent state. Gene Dev.

[bib1.bib10] Liu H, Zhu F, Yong J, Zhang P, Hou P, Li H, Jiang W, Cai J, Liu M, Cui K, Qu X (2008). Generation of induced pluripotent stem cells from adult rhesus monkey fibroblasts. Cell Stem Cell.

[bib1.bib11] Mahmoudi S, Brunet A (2012). Aging and reprogramming: a two-way street. Curr Opin Cell Biol.

[bib1.bib12] Marchetto MC, Narvaiza I, Denli AM, Benner C, Lazzarini TA, Nathanson JL, Paquola AC, Desai KN, Herai RH, Weitzman MD, Yeo GW (2013). Differential L1 regulation in pluripotent stem cells of humans and apes. Nature.

[bib1.bib13] Mattison JA, Vaughan KL (2016). An overview of nonhuman primates in aging research. Exp Gerontol.

[bib1.bib14] Mishra A, Qiu Z, Farnsworth SL, Hemmi JJ, Li M, Pickering AV, Hornsby PJ (2016). Induced pluripotent stem cells from nonhuman primates. Methods Mol Biol.

[bib1.bib15] Nichols J, Smith A (2009). Naive and primed pluripotent states. Cell Stem Cell.

[bib1.bib16] Nishijima K, Saitoh R, Tanaka S, Ohsato-Suzuki M, Ohno T, Kitajima S (2012). Life span of common marmoset (*Callithrix jacchus*) at CLEA Japan breeding colony. Biogerontology.

[bib1.bib17] Polo JM, Liu S, Figueroa ME, Kulalert W, Eminli S, Tan KY, Apostolou E, Stadtfeld M, Li Y, Shioda T, Natesan S (2010). Cell type of origin influences the molecular and functional properties of mouse induced pluripotent stem cells. Nat Biotechnol.

[bib1.bib18] Prigione A, Fauler B, Lurz R, Lehrach H, Adjaye J (2010). The Senescence-related mitochondrial/oxidative stress pathway is repressed in human induced pluripotent stem cells. Stem Cells.

[bib1.bib19] Qiu Z, Farnsworth SL, Mishra A, Hornsby PJ (2013). Patient-specific induced pluripotent stem cells in neurological disease modeling: the importance of nonhuman primate models. Stem Cells Cloning.

[bib1.bib20] Qiu Z, Mishra A, Li M, Farnsworth SL, Guerra B, Lanford RE, Hornsby PJ (2015). Marmoset induced pluripotent stem cells: Robust neural differentiation following pretreatment with dimethyl sulfoxide. Stem Cell Res.

[bib1.bib21] Quintanilla Jr RH (2013). Cellular characterization of human pluripotent stem cells. Methods Mol Biol.

[bib1.bib22] Ross CN, Davis K, Dobek G, Tardif SD (2012). Aging phenotypes of common marmosets (Callithrix jacchus). J Aging Res.

[bib1.bib23] Sardo VL, Ferguson W, Erikson GA, Topol EJ, Baldwin KK, Torkamani A (2017). Influence of donor age on induced pluripotent stem cells. Nat Biotechnol.

[bib1.bib24] Takahashi K, Yamanaka S (2006). Induction of pluripotent stem cells from mouse embryonic and adult fibroblast cultures by defined factors. Cell.

[bib1.bib25] Takahashi K, Tanabe K, Ohnuki M, Narita M, Ichisaka T, Tomoda K, Yamanaka S (2007). Induction of pluripotent stem cells from adult human fibroblasts by defined factors. Cell.

[bib1.bib26] Wu Y, Melton DW, Zhang Y, Hornsby PJ (2009). Improved coinfection with amphotropic pseudotyped retroviral vectors. BioMed Res-International.

[bib1.bib27] Wu Y, Zhang Y, Mishra A, Tardif SD, Hornsby PJ (2010). Generation of induced pluripotent stem cells from newborn marmoset skin fibroblasts. Stem Cell Research.

[bib1.bib28] Wunderlich S, Kircher M, Vieth B, Haase A, Merkert S, Beier J, Göhring G, Glage S, Schambach A, Curnow EC, Pääbo S (2014). Primate iPS cells as tools for evolutionary analyses. Stem Cell Research.

[bib1.bib29] Yu J, Vodyanik MA, Smuga-Otto K, Antosiewicz-Bourget J, Frane JL, Tian S, Nie J, Jonsdottir GA, Ruotti V, Stewart R, Slukvin II (2007). Induced pluripotent stem cell lines derived from human somatic cells. Science.

