# Supplementary material for: Overcoming barriers to reprogramming and differentiation in nonhuman primate induced pluripotent stem cells
Source: Primate Biol. 2017 Aug 18;4(2):153–62. doi: 10.5194/pb-4-153-2017 (PMC7041531; doi:10.5194/pb-4-153-2017)
Supplement: The supplement related to this article is available online at: https://doi.org/10.5194/pb-4-153-2017-supplement. [file pb-4-153-supplement.zip › pb-4-153-2017-supplement-title-page.pdf]

Supplement of Primate Biol., 4, 153–162, 2017
https://doi.org/10.5194/pb-4-153-2017-supplement
© Author(s) 2017. This work is distributed under
the Creative Commons Attribution 3.0 License.

Supplement of

Overcoming barriers to reprogramming and differentiation in nonhuman primate induced pluripotent stem cells

Jacob J. Hemmi et al.

Correspondence to: Peter J. Hornsby (hornsby@uthscsa.edu)

- pb-4-153-2017-supplement-title-page.pdf
- qPCR values.xlsx

The copyright of individual parts of the supplement might differ from the CC BY 3.0 License.
